# Genome-wide association study exploring the genetic architecture of eggshell speckles in laying hens

**DOI:** 10.1186/s12864-023-09632-7

**Published:** 2023-11-22

**Authors:** Xue Cheng, Xinghua Li, Mengyuan Yang, Chuanwei Zheng, Haiying Li, Lujiang Qu, Zhonghua Ning

**Affiliations:** 1 National Engineering Laboratory for Animal Breeding, College of Animal Science and Technology, China Agricultural University, Beijing, 100193 China; 2 Beijing Zhongnongbangyang Layer Breeding Co., Ltd, Beijing, 100083 China; 3 College of Animal Science, Xinjiang Agricultural University, Urumqi, 830000 China

**Keywords:** Laying hens, Eggshell speckles, Egg quality, GWAS

## Abstract

**Background:**

Eggshell speckle phenotype is an important trait in poultry production because they affect eggshell quality. However, the genetic architecture of speckled eggshells remains unclear. In this study, we determined the heritability of eggshell speckles and conducted a genome-wide association study (GWAS) on purebred Rhode Island Red (RIR) hens at 28 weeks to detect potential genomic loci and candidate genes associated with eggshell speckles.

**Results:**

The heritability of eggshell speckles was 0.35 at 28 weeks, and the speckle level is not related to other eggshell quality traits in terms of phenotypic correlation. We detected 311 SNPs (6 significantly, and 305 suggestively associated) and 39 candidate genes associated with eggshell speckles. Based on the pathway analysis, the 39 candidate genes were mainly involved in alpha-linolenic acid metabolism, linoleic acid metabolism, ether lipid metabolism, GnRH signaling pathway, vascular smooth muscle contraction, and MAPK signaling pathway. Ultimately, ten genes, *LOC423226, SPTBN5, EHD4, LOC77155, TYRO3, ITPKA, DLL4, PLA2G4B, PLA2G4EL5*, and *PLA2G4EL6* were considered the most promising genes associated with eggshell speckles that were implicated in immunoregulation, calcium transport, and phospholipid metabolism, while its function in laying hens requires further studies.

**Conclusions:**

This study provides new insights into understanding the genetic basis of eggshell speckles and has practical application value for the genetic improvement of eggshell quality.

**Supplementary Information:**

The online version contains supplementary material available at 10.1186/s12864-023-09632-7.

## Background

As a globally important poultry species, hens are widely farmed in China for egg production due to their high-quality protein and affordable price. Brown-shelled eggs are favoured by consumers in the Chinese market [1]. After extended periods of selection for eggshell colour, brown eggs have gained a dark and uniform colour. However, as a side effect, some brown eggs have speckles on their eggshell surface, which can considerably affect egg appearance and consumer preference. The selection against eggshell speckles in the breeding process can minimise the loss of egg value and increase the economic benefits for breeders.

**Fig. 1:**
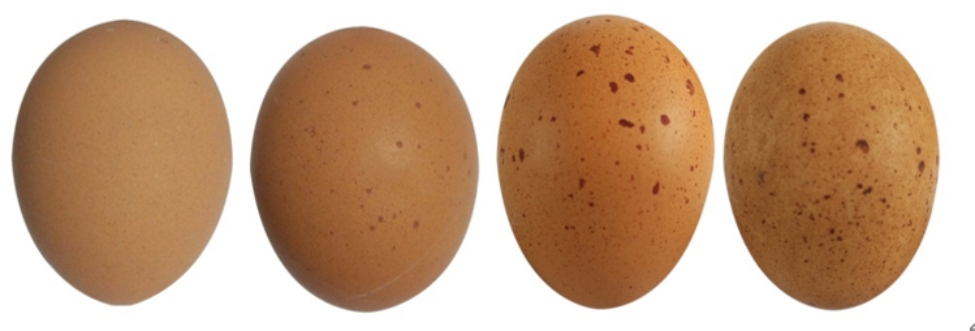
Recording eggshell speckles. Eggshell spot density increases from left to right, representing grades 1, 2, 3, and 4 [2, 4]

The speckle level can be recorded using a scoring method based on the severity of eggshell speckles artificially, as shown in Fig. 1 (scored in increments of 1, from 1 for no speckles to 4 for the most) [2]. This scoring method has also been widely used to assess the severity of speckling on eggs in passerine birds [3]. Previous studies have shown that speckles are widespread in brown-shelled eggs of laying hens, and that the proportion of speckled eggs laid increases with age [2]. It has been established that eggshell speckles are located between the vertical crystal layer and the cuticle layer of the eggshell [4], and they consist chiefly of protoporphyrin, which is the main component of eggshell pigment [3]. Previously, we reported that speckled eggs have a low hatchability which could lead to huge economic losses [4]. Therefore, understanding the genetic architecture of speckled eggs is important for breeders to establish adequate selection strategies to reduce the incidence of speckled eggs and improve eggshell quality.

The Rhode Island Red (RIR) hen, a pure line widely farmed in China, is also the representative of brown-shelled egg layers. The genetic parameters of speckled eggs in RIR chickens and the extent to which the speckle level is phenotypically and genetically related to eggshell quality remain unclear. Therefore, in the present study, we evaluated the genetic parameters of eggshell speckles and determined the phenotypic and genetic correlations between eggshell speckle levels and egg quality traits (including eggshell colour, eggshell shape, and egg weight). A genome-wide association study (GWAS) was employed to detect candidate genes related to eggshell speckles, which may be valuable for the genetic improvement of egg quality.

## Results

### Phenotype statistics and genetic parameters estimation

The eggshell quality and the speckle trait of the RIR hens are described in Table 1. The coefficient of variation (CV) of Egg shape index (ESI) and shell colour b* was the smallest, whereas the CV values of eggshell strength (ESS) and eggshell speckle level (SL) were the largest. Table 2 shows the heritability of eggshell qualities and SL of the hens 28 weeks old based on the pedigree. We found that the heritability of different egg qualities was 0.26–0.50, of which egg weight had the highest heritability, and the egg colour b* value had the lowest heritability. The heritability of eggshell speckles was 0.35, which indicates medium heritability.

**Table 1:** Egg quality description at 28 weeks

	N	Mean	SD	CV (%)	Min	Max
L*	1220	60.76	3.69	6.07	48.83	78.06
a*	1220	18.20	1.96	10.77	8.32	23.11
b*	1220	30.00	1.52	5.07	22.65	33.78
ESI	1220	1.30	0.04	3.08	1.17	1.47
EW (g)	1220	55.23	3.66	6.63	38.67	69.10
ESS (kg/cm^2^)	1220	4.45	0.61	13.71	2.02	6.91
SL	1220	1.15	0.48	41.74	1	4

**Note**: N: number; Mean: arithmetic mean; SD: standard deviation; CV: coefficient of variation; L*, a*, and b*: eggshell colour; ESI: egg shape index; EW: egg weight; ESS: eggshell strength; SL: speckle level

**Table 2:** The genetic parameters of eggshell quality traits

Trait	Heritability	SE
L*	0.46	0.07
a*	0.39	0.07
b*	0.26	0.06
ESI	0.37	0.07
EW	0.50	0.07
ESS	0.28	0.06
SL	0.35	0.07

**Note**: SE: standard error; L*, a*, and b*: eggshell colour; ESI: egg shape index; EW: egg weight; ESS: eggshell strength; SL: speckle level

**Fig. 2:**
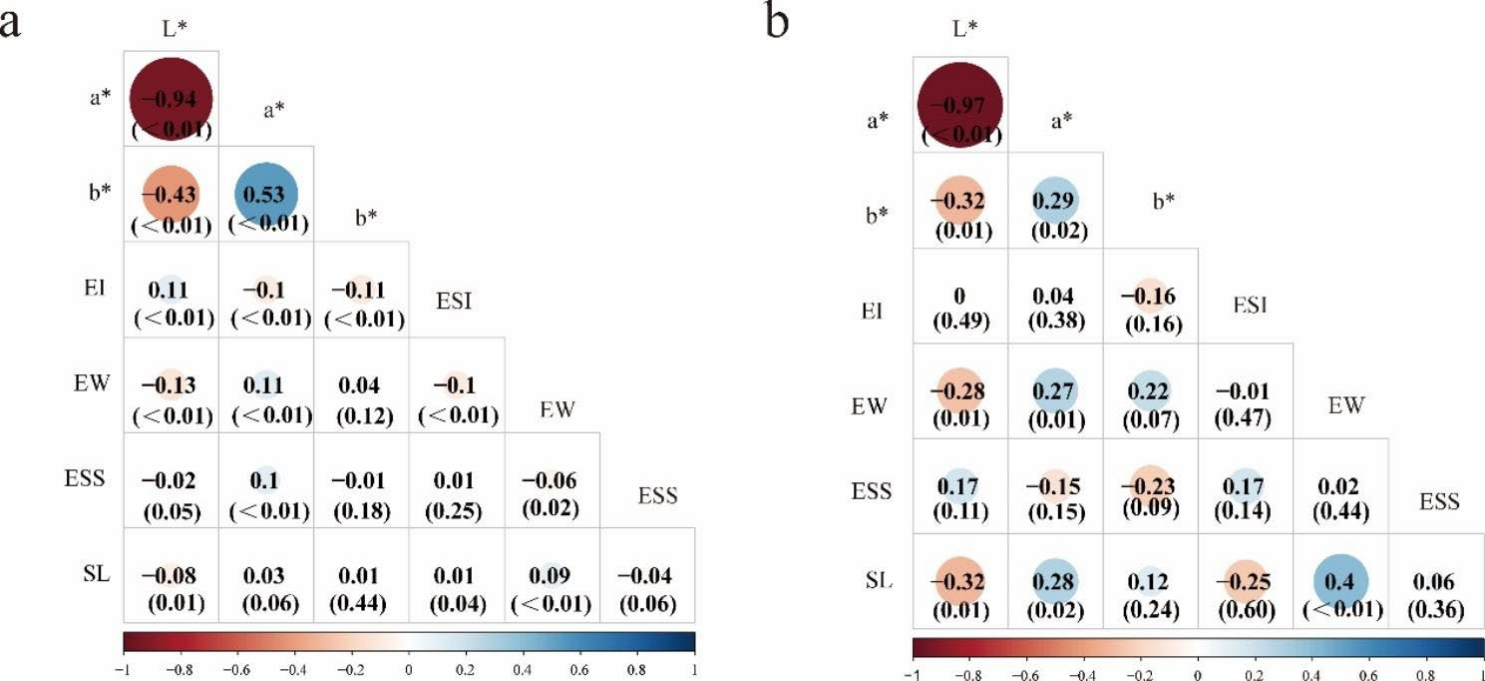
Genetic properties between eggshell speckles and quality. (**a**) Phenotypic correlations between eggshell speckle level and quality. (**b**) Genetic correlations between eggshell speckle level and quality. L*, a*, and b*: eggshell colour; ESI: egg shape index; EW: egg weight; ESS: eggshell strength; SL: speckle level. P values are in parentheses

The phenotypic correlations between SL and eggshell quality are shown in Fig. 2a. The eggshell colour L*, a*, and b* values were highly significantly correlated. The phenotypic correlation between SL and other eggshell qualities was very low, ranging from − 0.08 to 0.09. The eggshell colour L* value had a strong negative phenotypic correlation with the a* value, which was close to -1. The genetic correlation estimation of eggshell qualities with speckle level is shown in Fig. 2b. Moderately positive genetic correlations between SL and the eggshell colour a* value and egg weight (EW) were observed (the correlation coefficients were 0.28 and 0.4, respectively). The SL had a moderately negative genetic correlation with the eggshell colour L* value and ESI (the correlation coefficients were − 0.32 and − 0.25, respectively).

### Genome-wide association analyses

**Fig. 3:**
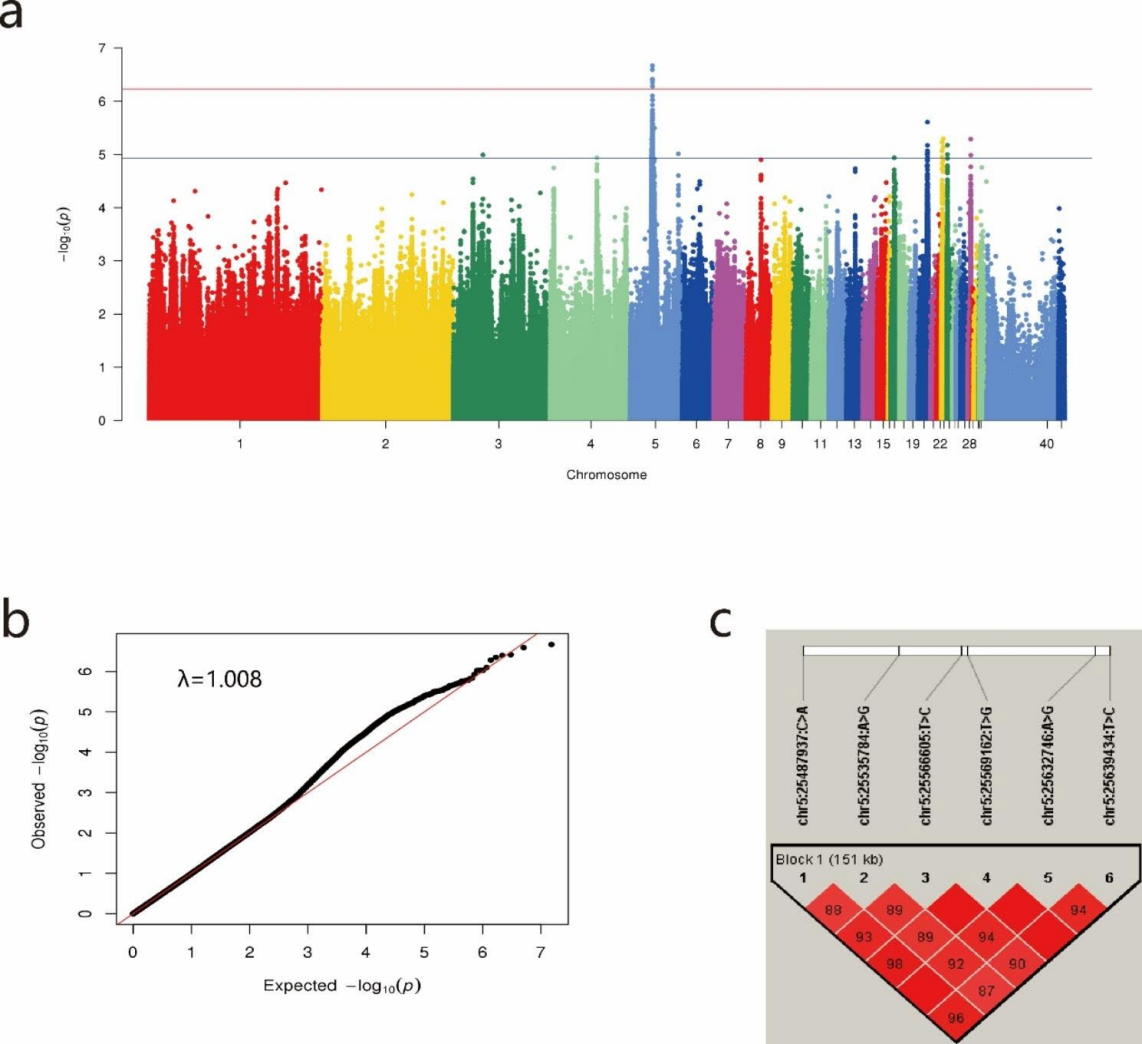
GWAS results of eggshell speckling. (**a**) Manhattan plot; (**b**) QQ plots; (**c**) LD plot for significant SNPs. In the Manhattan plot, the X-axis represents the chromosomal position corresponding to each SNP (40 and 41 represent the Z and W chromosomes, respectively). The Y-axis represents -log10 (P values). The red and blue lines represent the genome-wide significant threshold (5.87 × 10^− 7^) and suggestive significant threshold (1.17 × 10^− 5^), respectively. The QQ plot shows the expected -log10-transformed p value against the observed -log10-transformed p value. The λ value (1.008) is marked in the upper left of the QQ plot. The top line represents the position of the significant SNPs.

A total of 53 individuals laying normal eggs with an average score of 1 and 54 individuals laying speckled eggs with an average score of 2.5 or higher were selected as the control and case groups, respectively. After a series of strict quality control procedures, 107 individuals and 7,589,928 SNPs were used in the subsequent GWAS analysis. The Manhattan and Quantile–Quantile (QQ) plots are shown in Fig. 3a and b, respectively. The λ value in the QQ plots was 1.008, which was slightly higher than the ideal value of 1, indicating that population stratification was well controlled. Six SNPs and 305 SNPs were found to be significantly and suggestively significantly associated with eggshell speckle, respectively. Linkage disequilibrium (LD) analysis of significant SNPs was performed using Haploview software. The results showed that these six significantly associated SNPs were located in a block region (Fig. 3c), showing a strong linkage disequilibrium state, and it was difficult to identify the causal mutation.

The chi-square test was used to compare the allele frequencies of the six identified significant SNPs in the control and case groups. The results showed that the allele frequencies of the six significant SNPs differed significantly between the two groups (Supplementary Table S1).

### SNP annotation and pathway analyses

Using snpEFF software to annotate the SNPs, we found that the significantly associated SNPs were located in the intron of chromosome 5 (Table 3). Using the GEMMA software, we found that the significant SNP chr5: 25,535,784: A > G (P = 3.83 × 10^− 7^; intron variant) explained 7.80% of the phenotypic variance. The 305 suggestively significantly associated SNPs were mainly distributed on chicken chromosomes 5, 20, and 23, and the SNPs were mainly located in introns and intergenic regions, upstream and downstream of gene, corresponding to 39 genes (Supplementary Table S2). GO analysis of the 39 genes indicates that these genes play an important role in calcium-dependent phospholipase A2 activity, glycerophospholipid catabolic process, phospholipase A2 activity, and calcium-dependent phospholipid binding (Supplementary Table S3). These candidate genes are mainly enriched in the KEGG pathways including alpha-linolenic acid metabolism, linoleic acid metabolism, ether lipid metabolism, GnRH signaling pathway, vascular smooth muscle contraction, and MAPK signalling pathway (Supplementary Table S4).

**Table 3:** Genome-wide significant SNPs associated with eggshell speckles

SNP ID	CHR	Position (bp)	Effect allele	Other allele	Allele frequency	Beta	Se	P value	PVE (%)	Annotation	Gene Name
chr5: 25,569,162: T > G	5	25,569,162	G	T	0.42	-0.31	0.06	2.14 × 10^− 7^	4.27	Intron variant	*LOC423226*
chr5: 25,487,937: C > A	5	25,487,937	A	C	0.43	-0.31	0.06	2.56 × 10^− 7^	3.51	Intron variant	*SPTBN5*
chr5: 25,535,784: A > G	5	25,535,784	A	G	0.43	-0.29	0.05	3.83 × 10^− 7^	7.80	Intron variant	*EHD4*
chr5: 25,639,434: T > C	5	25,639,434	C	T	0.45	-0.31	0.06	3.99 × 10^− 7^	2.66	Intron variant	*LOC771552*
chr5: 25,566,605: T > C	5	25,566,605	C	T	0.42	-0.30	0.06	4.45 × 10^− 7^	2.54	Intron variant	*LOC423226*
chr5: 25,632,746: A > G	5	25,632,746	A	G	0.42	-0.31	0.06	5.22 × 10^− 7^	2.16	Intron variant	*LOC771552*

**Note**: PVE: percent phenotypic variation explained

## Discussion

The eggshell speckles of birds commonly serve as camouflage and play a role in thermoregulation. A previous study showed that the incidence of speckles in brown-egg lines may be associated with extreme selection for eggshell colour during breeding [5]. In the present study, we measured the phenotypic and genetic correlations between the speckle level and eggshell quality. Our results showed that SL had a moderate negative genetic correlation with the eggshell colour L* value and a moderate positive correlation with the a* value. This finding is consistent with that reported by Arango et al. [5]. They reported that extreme selection for a lower eggshell L* value and higher a* value resulted in more severe eggshell speckle levels. The L* value of eggshell colour indicates brightness. The higher the L* value, the lighter the eggshell colour. The a* value represents the colour range from green to red. The higher the a* value, the closer the eggshell colour is to red. Considering that the brown colour of both eggshells and speckles involves the deposition of the pigment protoporphyrin, it seems plausible that the selection for a darker eggshell colour would also lead to a higher incidence of speckles.

Furthermore, our results showed that SL has a moderate and positive genetic correlation but a low phenotypic correlation with EW, implying that heavier eggs tend to have more severe speckle levels. However, there have been no studies on the genetic correlation between SL and EW. Further studies are required to explore the relationship between these two traits.

The ASReml software was used to determine the heritability of eggshell speckles and other eggshell qualities based on the pedigree. The heritability of SL reported by J Arango [5] was 0.30 for the early laying stage (25 weeks of age) and 0.50 for the late laying state (40 weeks of age), which is consistent with the results obtained in the present study. The heritability of EW obtained in this study is similar to previously reported results ranging from 0.33 [6] to 0.71 [7]. The heritability of ESI and ESS estimated in previous studies [8, 9] was also consistent with results from the present study. The previously reported heritabilities for the eggshell colour, L* and b* values [10] are higher than those in the present study, which may be due to the differences in models, species, and ages of hens.

Although the heritability of eggshell speckles has been previously investigated and found to be moderate to low, the genomic architecture of the eggshell speckles remains unclear. GWAS is an effective way to identify candidate genes and SNPs, and has been widely used to analyse the genetic mechanisms of important economic traits in livestock [11–13]. In this study, we identified six SNPs significantly associated with eggshell speckles and four corresponding genes (*LOC423226*, *SPTBN5*, *EHD4*, and *LOC771552*) which were located on chromosome 5. The *SPTBN5* gene encodes the βV-spectrin protein in many tissues and is involved in maintaining the stability, structure, and shape of the cell membrane [14]. A variant of *SPTBN5* leads to intellectual disability, seizures, and developmental delays in humans [15]. The βV-spectrin protein is involved in the cell signalling pathway and immune response in chickens with viral respiratory infections [16]. Studies have reported that mutations in *SPTBN5* are related to adaptation to cold conditions and contribute to parasitic resistance in cattle [17, 18]. In this study, the SNP with the highest percent phenotypic variation explained (PVE) was chr5: 25,535,784: A > G, which is located in the intron region of the EH domain-containing 4 (*EHD4*) gene. *EHD4* has been described as a critical regulator of endocytic transport of cell membranes [19]. It has been demonstrated that the EHD protein plays a positive and important role in the autoimmune response in vivo and antigen-specific T-cell activation in vitro [20], and the endocytic recycling mediated by the EHD protein is involved in the development of germ cells [21]. *EHD4* is reportedly associated with endometritis in cows [22]. In our previous study, we found that differentially methylated genes between the hens laying speckled and unspeckled eggs are associated with immune-related genes and T-cell-mediated immunity pathways [23]. Based on our previous and current results, we speculate that the *EHD4* gene may participate in the regulation of T-cell-mediated immunity. Little is known about the function of the *LOC423226* and *LOC771552* genes in the formation of speckled eggs, and further investigation is needed.

We also identified other candidate genes associated with speckled eggshell, including *TYRO3, ITPKA, DLL4*, and some genes involved in the phospholipid metabolism pathway. *TYRO3* is part of a 3-member transmembrane receptor kinase receptor family. The encoded protein is involved in controlling cell survival and proliferation, immunoregulation, and phagocytosis [24–26]. It has been reported that *TYRO3* gene participates in the regulation of the immunity mechanism in the spleen of laying hens infected by bronchitis virus [27]. *ITPKA* regulates inositol phosphate metabolism and calcium signaling by phosphorylation of second messenger inositol-1,4,5-trisphosphate to inositol-1,3,4,5-tetrakisphosphate [28]. *ITPKA* has also been found differentially expressed in laying hens’ uterus and to be associated with calcium signalling pathway [29]. *DLL4* encodes a membrane-bound ligand and plays an important role in the control of endothelial cell proliferation, migration, and angiogenic sprouting [30, 31]. In the pathway analysis of all candidate genes, we found that candidate genes were mainly enriched in phospholipid metabolism-related pathways (*PLA2G4B, PLA2G4EL5, and PLA2G4EL*6). Phospholipids are important components of biofilms and regulate fat metabolism. *PLA2G4B* encodes phospholipase A2, which can participate in membrane homeostasis by changing phospholipid composition [32]. We speculated that immunoregulation, calcium transport, proliferation, migration of vascular endothelial cells, and phospholipid metabolism may affect the formation of eggshell speckles, but the detailed mechanism needs further study.

## Conclusions

In summary, we calculated the heritability of speckled eggs to be 0.35, indicating that speckling is a moderately heritable trait. A GWAS was used to reveal the genetic architecture of speckled eggs. We detected 311 SNPs (6 significantly, and 305 suggestively significantly associated) and 39 candidate genes associated with eggshell speckles. Based on the pathway analysis, the 39 candidate genes were mainly involved in alpha-linolenic acid metabolism, linoleic acid metabolism, ether lipid metabolism, GnRH signaling pathway, vascular smooth muscle contraction, and MAPK signaling pathway. Ultimately, ten genes, *LOC423226, SPTBN5, EHD4, LOC77155, TYRO3, ITPKA, DLL4, PLA2G4B, PLA2G4EL5*, and *PLA2G4EL6* were considered the most promising genes associated with eggshell speckles that were implicated in immunoregulation, calcium transport, and phospholipid metabolism, while its function in laying hens requires further studies. This study provides a reference for further exploring the molecular mechanisms underlying egg speckling.

## Methods

### Ethics statement

All chicken were obtained from Beijing Zhongnongbangyang Layer Breeding Co., Ltd (Beijing, China). This study was performed in accordance with the ethical and animal welfare guidelines approved by the ethics committee of China Agricultural University (permit number: AW11403202-1-32). All experimental protocols were performed according to the guidelines established by the Ministry of Science and Technology (Beijing, China). We declare that this study was reported in accordance with ARRIVE guidelines.

### Animals and data collection

We used a population of purebred RIR hens from Beijing Zhongnong Model Laying Hens Breeding Co., LTD (Beijing, China) as the experimental animals. The RIR chicken is a commercial brown-shell layer breed that has been selected for production performance over many years. This study included 1266 pedigreed hens aged 28 weeks. All birds were raised in individual cages under identical environmental and management conditions. The temperature of the housing was controlled at 20 ± 1 °C and the lighting program comprised a 16 / 8 h light–dark cycle. The experimental chickens had free access to drinking water and were fed at a fixed time every day. All birds were free of *Salmonella pullorum* and avian leukosis viruses.

Eggs were collected from all hens for quality measurements. The measurement indices included EW, Eggshell colour (ESC), ESI, ESS, and SL. An electronic balance was used to determine EW. An eggshell colour tester (CM-2600d, Konica Minolta Optics Inc, Tokyo, Japan), which works on the L* (brightness), a* (green-red), and b* (blue-yellow) colour model, was used to measure the ESC. The ESI value (length/breadth) was measured using Vernier callipers. An Eggshell Force Gauge (Model II, Robotmation Co. Ltd., Tokyo, Japan) was used to assess the ESS. Finally, SL was determined using a previously published scoring method [4]. A level of one indicates an eggshell without speckles, and levels 2, 3, and 4 indicate increasing levels of speckles on the eggshell (Fig. 1) [2, 4].

We recorded the SL of each egg for the first five days. On the sixth day, individuals laying level 1 eggs were grouped into the control group, and those laying eggs at levels 3 and 4 were grouped into the case group. Finally, we selected 54 and 53 hens from the case and control groups, respectively. Blood samples were collected from the wing vein and stored in anticoagulant blood collection vessels at -20 °C for subsequent DNA extraction.

### Estimation of genetic parameters

Phenotypic and genetic correlations and heritabilities were estimated using ASReml software based on the pedigree information. Multiple trait model was used to estimate phenotypic and genetic correlations and heritabilities. The model used for each trait was as follows:

$${\rm{y}}\,{\rm{ = }}\,{\rm{Xb}}\,{\rm{ + }}\,{\rm{Za}}\,{\rm{ + }}\,{\rm{e}}$$

where y is the vector of the daily speckle level; b is the vector of fixed effects; e is the vector of random residuals; and X and Z are the incidence matrices of the fixed and genetic effects, respectively.

### Genomic sequencing, imputation, and quality control

Genomic DNA was extracted from the blood using a TIANamp Blood DNA Kit (Tiangen, Beijing, China). DNA concentration and purity were assessed using a NanoDrop 2000 spectrophotometer (Thermo Fisher Scientific, Waltham, MA, USA). The qualified DNA samples were sent to a commercial company for next-generation sequencing. All libraries were sequenced on the MGI DNBSEQ T7 platform with 150 bp paired-end reads. The sequence coverage of each sample was 10 ×.

Low-quality reads and adapter sequences were removed using the fastp (version 0.20.1) software [33] with the following parameters: –length_required 30, –trim_front1 10, and –trim_front2 10. The clean data were aligned to the chicken reference genome (galGal6a) using BWA (version 0.7.17) [34] with default parameters and sorted using samtools (version 1.10) [35]. Individual SNPs and INDEL files were selected using the GATK (version 3.8) HaplotypeCaller tool and combined using the GATK combineGVCFs tool [36]. The SNPs of the population were selected and filtered using the GATK SelectVariants and VariantFiltration tools with the following cut-off values: QD < 2.0, MQ < 40.0, and FS > 60.0. The passed SNPs were filtered using PLINK (version 1.9) [37] with the following parameters: –geno 0.05, –maf 0.05, and –hwe 0.000001. Beagle (version 4.0) [38] was used to impute the SNPs and individuals that met the criteria. After imputation, PLINK (version 1.9) was used for secondary quality control with the following parameters: –mind 0.05, –geno 0.05, –maf 0.05, and –hwe 0.000001, and the final number of effective SNPs and individuals was 7,589,928 and 107, respectively. Subsequent analyses were based on this SNP dataset.

### Genome-wide association analyses

GWAS of the eggshell speckle trait was performed using the univariate mixed linear model in the GEMMA software [39], with the first three principal components as covariates. The model used to analyse each SNP was as follows:

$${\rm{y}}\,{\rm{ = }}\,W\alpha \,{\rm{ + }}\,x\beta \,{\rm{ + }}\,{\rm{u}}\,{\rm{ + }}\,\varepsilon$$

where y represents the individual phenotypic value, namely either ‘0’ or ‘1’; W refers to the covariance matrix, the first column of which is the column vector 1, the second to the fourth columns are the fixed effects, the top three principal components respectively; 𝛼 denotes a vector comprising the intercept and corresponding coefficient; 𝑥 denotes a vector of the SNP genotypes; 𝛽 refers to the effects of the corresponding SNP; 𝑢 is a vector of random effects, obeying the covariance structure: u ~ N (0, KVg) normal distribution, which is suitable for the genetic relationship matrix K, Vg is the multiple gene additive variance; and 𝜀 is a vector of random residuals. The SimpleM package was used in this study to calculate the number of effective independent tests [40]: 85,137 effective independent tests were obtained. Therefore, the significance threshold was set as 5.87 × 10^− 7^ (0.05/85,137), and the suggestive significance threshold was set as 1.17 × 10^− 5^ (1/85,137).

The R “qqman” package was used to draw Manhattan and QQ plots. The genomic inflation factor λ was calculated using the estlambda function of the GenABEL software package in R [41]. For significant SNPs associated with the speckle trait, the GEMMA software was used to estimate their contribution to phenotypic variance.

### Linkage disequilibrium analysis and candidate gene identification

LD analysis of significant SNPs was performed using the Solid Spine algorithm in Haploview software (version 4.2) [42]. The snpEFF software [43] was used to annotate significantly associated SNPs, and the biological functions of those candidate genes were checked using PubMed (https://www.ncbi.nlm.nih.gov/pubmed). KOBAS [44] (http://kobas.cbi.pku.edu.cn/) for GO and KEEG analyses.

### Statistical analysis

Eggshell quality was recorded and analysed using Microsoft Excel. The phenotypic and genetic correlation plot was drawn using corrplot in RStudio (version 1.3). Chi-square tests were performed using RStudio (version 1.3) to compare allele frequencies of four significant SNPs in the case and control groups. Haploview software (version 4.2) was used to draw LD plots.

### Electronic supplementary material

Below is the link to the electronic supplementary material.

**Supplementary Table S1**. The allele frequencies of six significant SNPs in the case and control groups

**Supplementary Table S3**. Gene Ontology enrichment analysis of candidate genes

**Supplementary Table S2**. Details of candidate SNPs associated with eggshell speckles

**Supplementary Table S4**. KEGG pathways analysis of candidate genes

## Data Availability

Raw sequencing data were uploaded to the NCBI BioProject (BioProject accession numbers: PRJNA964576).

## Abbreviations

GWAS: Genome-wide association study
RIR: Rhode Island Red
CV: Coefficient of variation
LD: Linkage disequilibrium
EW: Egg weight
ESC: Eggshell colour
ESI: Egg shape index
ESS: Eggshell strength
SL: Speckle level
