# Supplementary material for: Genome-wide association study exploring the genetic architecture of eggshell speckles in laying hens
Source: BMC Genomics. 2023 Nov 22;24:704. doi: 10.1186/s12864-023-09632-7 (PMC10666442; doi:10.1186/s12864-023-09632-7)
Supplement: Supplementary file 1 — Supplementary Table S1. The allele frequencies of six significant SNPs in the case and control groups [file 12864_2023_9632_MOESM1_ESM.docx]

**Supplementary Table S1.** The allele frequencies of six significant SNPs in the case and control groups

| CHR | SNP_ID | Group | Effect Allele Frequence | Other Allele Frequence | χ^2^  (p-vaule) |
| --- | --- | --- | --- | --- | --- |
| 5 | chr5: 25569162: T>G | Normal | 41.51% | 58.49% | 12.120  (<0.01) |
|  |  | Speckle | 75.00% | 25.00% |  |
| 5 | chr5: 25487937: C>A | Normal | 42.45% | 57.55% | 10.294  (<0.01) |
|  |  | Speckle | 71.30% | 28.70% |  |
| 5 | chr5: 25535784: A>G | Normal | 40.57% | 59.43% | 11.640  (<0.01) |
|  |  | Speckle | 73.15% | 26.85% |  |
| 5 | chr5: 25639434: T>C | Normal | 39.62% | 60.38% | 10.223  (<0.01) |
|  |  | Speckle | 70.37% | 29.63% |  |
| 5 | chr5: 25566605: T>C | Normal | 43.40% | 56.60% | 10.397  (<0.01) |
|  |  | Speckle | 73.15% | 26.85% |  |
| 5 | chr5: 25632746: A>G | Normal | 43.40% | 56.60% | 9.120  (<0.01) |
|  |  | Speckle | 72.22% | 27.78% |  |
